# Effects of Microbes on Insect Host Physiology and Behavior Mediated by the Host Immune System

**DOI:** 10.3390/insects16010082

**Published:** 2025-01-15

**Authors:** Shan Zhang, Zhengyan Wang, Qiong Luo, Lizhen Zhou, Xin Du, Yonglin Ren

**Affiliations:** 1 School of Food and Strategic Reserves, Henan University of Technology, Zhengzhou 450001, China; 2022920144@stu.haut.edu.cn (S.Z.); zywangedu@163.com (Q.L.); lizhenzhou@haut.edu.cn (L.Z.); 2 College of Environmental and Life Sciences, Murdoch University, Murdoch, WA 6150, Australia; b.du@murdoch.edu.au (X.D.); y.ren@murdoch.edu.au (Y.R.)

**Keywords:** microbe, immune system, growth and development, reproduction, pesticide resistance, chemical communication, metabolic reprogramming

## Abstract

Innate immunity is critical for insects to adjust to complicated environments. Studying the insect immune system can aid in identifying novel insecticide targets and provide insights for developing novel pest control strategies. Insects recognize environmental pathogens through pattern recognition receptors, thus activating the innate immune system to eliminate pathogens. The innate immune system of insects primarily comprises cellular immunity and humoral immunity. Toll, immune deficiency, and Janus kinase/signal transducers and activators of transcription are the main signaling pathways regulating insect humoral immunity. Nevertheless, increasing research has revealed that immune signaling activated by microbes also performs non-immune roles while exerting immune roles, and insulin signaling performs a key role in mediating the connection between the immune system and non-immune physiological activities. Therefore, this paper first briefly reviews the main innate immune signaling and insulin signaling of insects, then summarizes the relationship between immune signaling activated by microbes and insect growth and development, reproduction, pesticide resistance, chemical communication, cell turnover, lifespan, sleep, energy generation pathways and their possible underlying mechanisms. Future research directions and methodologies are also proposed, aiming to provide insights into further study on the physiological mechanism linking microbes and insect hosts.

## Simple Summary

Insects live in an environment enriched with microbes and thus have an evolved, complex immune system that recognizes pathogens and combats bacterial infections. In addition to immune functions, increasing research has revealed that the insect immune system also plays non-immune roles and insulin signaling plays an important role in mediating the relationship between the immune system and non-immune physiological activities. Major immune signaling activated by microbe infection, such as Toll, IMD, and JAK/STAT, participates in regulating insect growth and development, reproduction, pesticide resistance, and chemical communication by affecting insulin signaling. Additionally, activation of IMD may regulate insect oviposition behavior by affecting the nervous system, regulate insect resistance to phosphine by upregulating the expression of DUOX, regulate insect lifespan, and mediate the metabolic reprogramming of immune cells induced by hypoxia-inducible factor-1α. Furthermore, the activation of IMD and JAK/STAT can promote insect cell turnover.

## 1. Introduction

Insects possess an immune system that protects them from attacks by various pathogens that would otherwise threaten their survival. The immune system can combat pathogens by either eliminating them from the insect host or disarming them through the suppression of toxin and virulence factor synthesis, which are essential for pathogen invasion and destructive actions within the host [1]. The innate immune system of insects generally encompasses humoral responses and cellular responses [2]. Toll, immune deficiency (IMD), and Janus kinase/signal transducers and activators of transcription (JAK/STAT) are major signaling pathways responsible for activation of immune response in insects [1]. Insects recognize various pathogens in their environment through pattern recognition receptors (PRRs), which activate immune signaling pathways to regulate the production of immune effectors to eliminate pathogens. However, increasing evidence suggests that immune signaling pathways activated by microbes also perform non-immune functions, affecting various physiological and behavioral aspects of insects, including growth and development, reproduction, chemical communication, cell turnover, lifespan, and sleep [3,4,5].

Immune responses can suppress non-immune physiological activities. For instance, during pathogen infection, immune responses can lead to decreased overall metabolic rate, slowed growth and development, extensive depletion of energy reserves, and reduced reproductive capacity. These inhibitory effects can be explained by the physiological trade-off theory, which posits that one biological trait negatively impacts another within an individual [6]. According to this theory, both immune responses and non-immune physiological activities require energy that is limited. When the immune system is activated, it takes precedence over other processes in terms of energy supply [7]. On the other hand, immune responses can also promote certain non-immune physiological activities in insects, such as cell apoptosis and proliferation, chemical communication, and lifespan [3,4]. In practice, whether inducing inhibitory or promotive effects, insects rely on the interplay between immune and metabolic signaling pathways including insulin signaling to transmit information within or between cells and even between tissues, achieving regulation of overall metabolism when switching between immune responses and non-immune physiological activities [7].

Although much research has been conducted on insect immune systems, studies on the non-immune roles of immune signaling are relatively limited, and the physiological mechanisms involved are still lacking. For example, research on the metabolic reprogramming of immune cells and a systemic metabolic switch in insects is less advanced compared to mammals [7]. Additionally, the physiological mechanisms by which immune activation affects insect oogenesis remain unclear [6]. Further research on the non-immune effects of immune signaling will not only enhance our understanding of the multifaceted roles of the immune system within organisms but also help identify new insecticide targets, providing new strategies for pest control. For instance, understanding how the insect immune system affects its *Bacillus thuringiensis* (Bt) resistance could lead to the discovery of novel resistance mechanisms and the development of effective integrated pest management techniques [8].

## 2. Major Immune and Insulin Pathway Signaling in Insects

### 2.1. Toll Signaling

**Figure 1:**
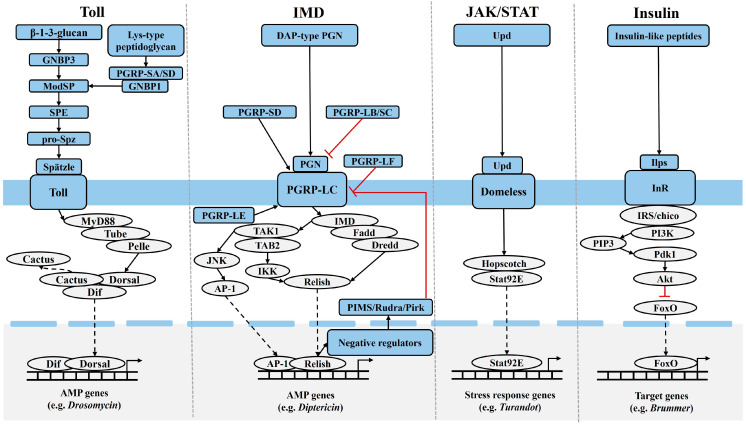
Major immune and insulin pathway signaling in insects. Black arrows depict activation while red bars represent suppression in molecular interactions. Black dashed bars represent nuclear translocation of transcription factors. Akt, protein kinase B; AP-1, activating protein-1; Brummer, lipase gene; Cactus, a nuclear inhibitory factor; DAP-type-PGN, meso-diaminopimelic acid-type peptidoglycan; Diptericin, antimicrobial peptide gene; Domeless, DNA adhesion transcription protein; Dorsal/Dif, a transcription factor; Dredd, death related ced-3/nedd2-like protein; Drosomycin, antifungal peptide gene; Fadd, fas-associated death-domain-containing protein; FoxO, fork head transcription factor O; GNBP, Gram-negative bacteria-binding protein; Hopscotch, a JAK tyrosine kinase; IKK, inhibitor of NF-κB kinase; Ilps, insulin-like peptides; IMD, immune deficiency; InR, insulin receptor; IRS/chico, insulin receptor substrate; JAK/STAT, Janus kinase/signal transducers and activators of transcription; JNK, c-Jun N-terminal kinase; ModSP, modular serine protease; MyD88/Tube/Pelle, plasmosin; Pdk1, phosphoinositide-dependent protein kinase 1; PGRP, peptidoglycan recognition protein; PI3K, phosphatidylinositol 3-kinase; PIMS/Rudra/Pirk, PGRP-LC-interacting inhibitors of IMD signaling; PIP3, phosphatidyl inositol triphosphate; pro-Spz, pro-Spätzle; Relish, a transcription factor; Spätzle, extracellular cytokine; SPE, Spätzle-processing enzyme; Stat92E, a transcription factor; TAB2, TAK1-binding protein 2; TAK1, transforming growth factor-β-activated kinase 1; Toll, Toll protein; Turandot, stress response genes; Upd, Unpaired.

Lysine-type peptidoglycan (PGN) produced by Gram-positive bacteria and β-1-3-glucan produced by fungi, respectively, bind to PGN recognition proteins (PGRPs) and Gram-negative binding proteins in hemolymph [9]. This binding activates a serine protease cascade, which facilitates the proteolytic cleavage of the inactive Spätzle precursor, leading to its activation. The activated Spätzle then binds to the Toll receptor, forming a Toll dimer. The activated Toll receptor interacts with the cytoplasmic myeloid differentiation factor 88 (MyD88)/tandem ubiquitin-binding entity (Tube)/Pelle trimer, resulting in Pelle phosphorylation. Phosphorylated Pelle dissociates from the trimer, transmitting the signal downstream to the nuclear inhibitor Cactus. Cactus inhibits the nuclear translocation of nuclear factor-κB (NF-κB)/Relish proteins, such as the transcription factors dorsal-related immunity factor (Dorsal/Dif), by sequestering them in the cytoplasm. Upon ubiquitination and phosphorylation of Cactus, Dorsal or Dif is released from the Cactus–Dorsal or Cactus–Dif complex and translocates into the nucleus. Once in the nucleus, they bind to κB-DNA elements in the promoter regions of target genes, initiating the expression of antimicrobial peptides (AMPs) [1] (Figure 1).

### 2.2. IMD Signaling

Diaminopimelic acid-type PGN produced by Gram-negative bacteria and bacilli binds to PRRs such as the transmembrane PGN recognition protein LC (PGRP-LC), activating IMD signaling. This activation subsequently triggers two downstream branches of IMD signaling: the Relish branch and the c-Jun N-terminal kinase (JNK) branch [10]. Activated Relish translocates into the nucleus to initiate the transcription of immune-related genes. Extracellular PGN recognition proteins SD (PGRP-SD) and LE (PGRP-LE) enhance the binding of PGN to PGRP-LC, thereby promoting PGN-mediated activation of IMD signaling [11,12]. IMD signaling diverges at the transforming growth factor β-activated kinase 1/TAK1-binding protein 2 (TAK1/TAB2) complex to form the JNK branch. JNK is a member of the mitogen-activated protein kinase (MAPK) family. TAK1, a mitogen-activated protein kinase kinase kinase (MAP3K), activates JNK through mitogen-activated protein kinase kinase 4 and hemipterous (Hep, homologous to mitogen-activated protein kinase kinase 7), which in turn activates activating protein-1 (AP-1). AP-1 then translocates into the nucleus to activate the transcription of stress response genes [10,13] (Figure 1).

Overactivation of IMD signaling can be harmful. To ensure normal growth and development and reproduction, insects have evolved corresponding negative regulatory mechanisms. For example, PIMS/Rudra/Pirk (PGRP-LC-interacting inhibitors of IMD signaling) acts as negative regulators of IMD signaling. In cultured cells and all immune tissues in vivo (haemocytes, fat body, and adult midgut), activated MAPK signaling can induce the expression of PIMS/Rudra/Pirk [14]. PIMS prevents PGRP-LC from migrating to the cell surface, reducing the number of PRRs on the cell membrane, thereby inhibiting the activation of IMD signaling. Both PGRP-LB and PGRP-SC possess amidase activity, which allows them to inhibit IMD signaling by degrading PGN [2]. PGRP-LF competitively binds to PGRP-LC, blocking its recognition of PGN [11] (Figure 1).

### 2.3. JAK/STAT Signaling

JAK-STAT signaling primarily participates in the antiviral immune response in insects. This signaling pathway is not directly triggered by viral ligands but is instead induced by the cytokine Unpaired (Upd), which is released by insect cells during infection. After viral nucleic acids are recognized by PRRs of host cells, NF-κB, interferon regulatory factors and other transcription factors are activated, leading to the expression of inflammatory cytokines, interferons, and other immune-related cytokines [15]. After cytokines released into the extracellular space bind to the transmembrane receptor Domeless, the tyrosine kinase JAK bound to the receptor protein phosphorylates Hopscotch. Activated Hopscotch then phosphorylates the transcription factor Stat92E, forming a homodimer that translocates into the nucleus to regulate the expression of downstream effector genes [16]. Cytokine-mediated JAK/STAT signaling is functionally conserved and plays an indispensable role in cell survival, proliferation, turnover, differentiation, apoptosis, and immune regulation [1] (Figure 1).

### 2.4. Insulin Signaling

Insulin/insulin-like signaling (IIS) regulates insect growth and development, reproduction, and aging by modulating carbohydrate, protein, and lipid metabolism [17]. Research on the insect IIS primarily focuses on phosphatidylinositol 3-kinase (PI3K)/protein kinase B (PKB, also known as Akt) signaling. Extracellular insulin binds to the insulin receptor (InR) on the cell membrane, leading to InR phosphorylation and the transmission of the insulin signal into the cell. Once inside the cell, IIS is primarily propagated via PI3K/Akt signaling [18]. Phosphorylated InR activates the InR substrate (IRS), which, upon binding with PI3K, generates phosphatidyl inositol triphosphate (PIP3) and recruits phosphoinositide-dependent protein kinase 1 (Pdk1) and Akt to the cell membrane. Pdk1 phosphorylates Akt at the Thr342 site [19]. The phosphorylated Akt then moves into the cytoplasm, where it regulates the activity of downstream genes such as the fork head transcription factor O (FoxO) family and glycogen synthase kinase-3, exerting its regulatory functions [20] (Figure 1).

## 3. Effects of Microbes on Insects

### 3.1. Effects of Microbes on Insect Growth and Development

**Figure 2:**
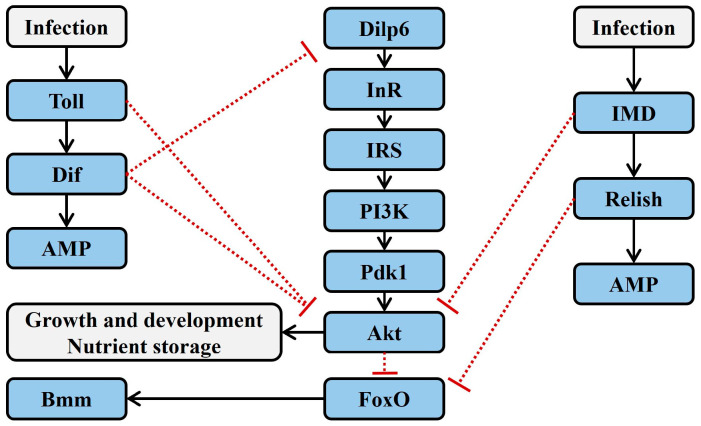
Known signaling interactions between immune and insulin signaling pathways. Black arrows depict known activation while red bars represent known suppression in molecular interactions. Red dashed bars represent known suppression between immune and IIS pathways, but the specific molecular interaction is unknown. Akt, protein kinase B; AMP, antimicrobial peptide; Bmm, brummer; Dif, a transcription factor; Dilp6, Drosophila insulin-like peptide 6; FoxO, fork head transcription factor O; IMD, immune deficiency; InR, insulin receptor; IRS, insulin receptor substrate; Pdk1, phosphoinositide-dependent protein kinase 1; PI3K, phosphatidylinositol 3-kinase; Toll, Toll protein.

There is a physiological trade-off between growth and development and immunity. Pathogen infection or genetic manipulation that activates the immune system of *Drosophila melanogaster* can lead to inhibited growth and reduced energy reserves. For example, chronic infection with the Gram-positive bacterium *Enterococcus faecalis* inhibits larval growth in *Drosophila* [21]. In *Drosophila R4/IMDCA* mutant strains, where IMD and Toll receptors are constitutively activated exclusively in the fat body, larvae exhibit prolonged developmental periods, decreased total triglyceride content, reduced lipid storage in the fat body, and lower body weight [22,23]. Further transcriptomic studies have confirmed that pathogen infection affects the expression of genes related to carbohydrate and lipid metabolism [24], and the overall level of Akt phosphorylation is reduced in adult *D. melanogaster* infected with *Mycobacterium marinum* [25]. Since IIS is a critical regulator of carbohydrate and lipid metabolism and is essential for normal insect growth and development, it is hypothesized that infection-induced shifts in carbohydrate and lipid metabolism could be mediated by alterations in IIS activity driven by the immune system [5] (Figure 2).

Toll signaling can inhibit insect growth and decrease triglyceride storage by suppressing Akt phosphorylation. In the fat body of larvae from the *D. melanogaster R4/IMDCA* mutant strain, constitutive activation of Toll signaling leads to reduced Akt phosphorylation in larval extracts. Furthermore, in *Drosophila* mutants with a loss of MyD88 function, the inhibition of Akt phosphorylation is reversed. These two studies complementarily verify the link between Toll and Akt signaling [22]. In larval fat body cells where PI3K is constitutively activated, the expression of Toll^10b^ (a Toll-like receptor) can block cell growth without affecting the production of PIP3, indicating that Toll signaling inhibits IIS downstream of IRS/chico and PI3K. Activated Toll signaling inhibits Akt phosphorylation at the Thr342 site by Pdk1, leading to reduced growth and triglyceride storage in *Drosophila* [19] (Figure 2).

Beyond interfering with Akt phosphorylation, Toll signaling can also regulate IIS by affecting the synthesis of insulin-like peptide (Ilp). Activating Toll signaling in *Drosophila* through genetic manipulation leads to reduced production of *Drosophila* insulin-like peptide 6 (Dilp6), which results in inhibited growth and development [26]. Infection with *E. faecalis* in *Drosophila* larvae activates Toll signaling and significantly decreases the overall content of Dilp6. Overexpression of *Dif* in the larval fat body can reduce Dilp6 mRNA transcripts and inhibit growth, and the infection-induced reduction in Dilp6 and growth suppression can be restored by RNAi knockdown of *Dif* [21]. However, whether Dif directly regulates Dilp6 expression or whether IIS is indirectly regulated by proteins directly controlled by Dif remains to be confirmed by further experiments [5] (Figure 2).

The overall growth inhibition in insects driven by Toll signaling in the fat body may also involve the induction of peripheral insulin resistance. Insulin resistance refers to the decreased sensitivity of target organs to insulin. Although activated Toll signaling can inhibit systemic growth in *Drosophila* by reducing Dilp6 in the fat body, it does not alter *Drosophila* insulin-like peptide 2 (Dilp2) content in the hemolymph (Dilp2 is an InR ligand secreted by insulin-producing cells in the brain). Constitutive activation of Toll^10b^ in the *Drosophila* larval fat body does not affect Dilp2 content in the hemolymph [27]. In *Mus musculus*, loss of IIS in adipose tissue leads to insulin resistance in liver and muscle [28,29]. It is therefore hypothesized that the downregulation of insulin synthesis in the fat body by Toll signaling may induce a slowdown in overall insect growth through a mechanism similar to mammalian insulin resistance [19].

Besides Toll signaling, IMD signaling can also influence insect growth and development. Although the activation of IMD and Toll signaling in *Drosophila* infected with *Escherichia coli* reduces Akt phosphorylation, this inhibition of Akt phosphorylation is reversed in Toll signaling mutants upon *E. coli* infection. This indicates that when both Toll and IMD signaling are activated simultaneously, Toll signaling inhibits IIS, while IMD signaling does not. Further, constitutive expression of *Relish* in the fat body of *Drosophila* does not significantly affect Akt phosphorylation or triglyceride content [22], but constitutive expression of IMD in the fat body significantly reduces systemic phosphorylation of Akt and ribosomal S6 kinase (S6K), delays larval growth and development, and decreases triglyceride content [23]. These opposing findings may be attributed to the use of different *Drosophila* genotypes in the constitutive activation of IMD signaling. It is therefore hypothesized that IMD signaling may have branches, where the main pathway activates Relish, while branches may regulate IIS through interactions with other pathways [5]. Additionally, while *Relish* knockdown in the fat body of *Drosophila* does not affect Akt phosphorylation, triglyceride, or glucose content, *Relish* knockdown in the enteroendocrine cells of the midgut suppresses Dilp3 transcription, increases intracellular lipid content, and decreases Akt phosphorylation [30]. This suggests that the regulation of IIS by IMD signaling has tissue-specific effects (Figure 2).

Insect FoxO can respond to various stresses, such as starvation, extreme temperatures and humidity, hypoxia, and pathogen infections, by regulating processes such as lipolysis and autophagy. Even in the absence of pathogen infection, starvation can induce FoxO-dependent expression of AMPs in the gut and fat body of *D. melanogaster* that is independent of Toll and IMD signaling [5]. FoxO can also activate the transcription of the lipase gene *brummer* (*bmm*), promoting lipolysis [31]. Under starvation conditions, wild-type *Drosophila* do not show a decrease in triglyceride content, while *Relish*-deficient *Drosophila* exhibit a significant reduction in triglyceride content. This is due to the upregulation of FoxO-dependent *bmm* transcription, indicating that IMD signaling can influence lipid metabolism by regulating FoxO activity. Further research finds that Relish can bind to Bmm, thereby inhibiting FoxO-dependent lipid degradation to prevent lipid depletion during starvation [5]. Future experiments are needed to verify whether the Relish–FoxO antagonism mediates changes in insect lipid metabolism during pathogen infection [25,32] (Figure 2).

### 3.2. Effects of Microbes on Insect Reproduction

**Figure 3:**
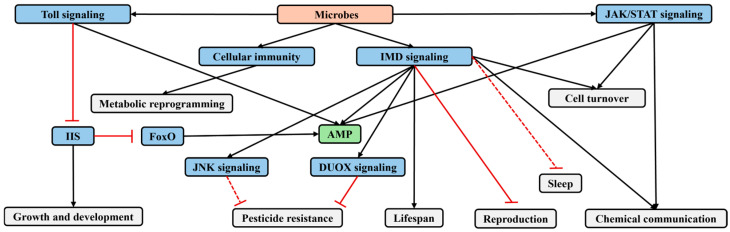
A generalized mechanism for the impacts of microbes on insect hosts mediated by the immune system. Black arrows depict activation while red bars represent suppression in molecular interactions. Red dashed bars represent that it is not determined whether it is a promoting or an inhibitory effect. AMP, antimicrobial peptide; DUOX, dual oxidase; FoxO, fork head transcription factor O; IIS, insulin/insulin-like signaling; IMD, immune deficiency; JAK/STAT, Janus kinase/signal transducers and activators of transcription; JNK, c-Jun N-terminal kinase.

There is a physiological trade-off between reproduction and immunity, with immune activation often leading to reduced reproductive capacity in insects (Figure 3). For instance, injection of *E. coli* or *Beauveria bassiana* into female *D. melanogaster* activates their immune response, resulting in a sustained decrease in reproductive output to 45% of baseline levels [33]. Similarly, feeding female *Drosophila* with a diet containing *Providencia rettgeri* triggers an immune response and decreases reproductive capacity [34]. Furthermore, activation of the immune response in *Drosophila* using various doses of crude lipopolysaccharides from *Serratia marcescens* leads to a dose-dependent reduction in female reproductive output [35]. The activity of IIS can be used to determine whether female insects have sufficient nutritional reserves for oogenesis [6]. High IIS activity promotes oogenesis, and under reduced IIS activity (indicator of nutritional deprivation) egg production is reduced or halted [36]. Therefore, it can be inferred that pathogen infection may inhibit oogenesis in insects by suppressing IIS [6]. However, this regulatory mechanism requires further rigorous experimental validation.

Furthermore, activated IMD signaling may also regulate insect oviposition behavior by affecting the nervous system (Figure 3). Studies have shown that neurons in the insect brain can activate IMD signaling in response to sensing PGN, thereby inhibiting female oviposition behavior; additionally, octopaminergic neurons in the insect brain can express the endogenous PGN-degrading enzyme PGRP-LB [37]. It is hypothesized that extracellular negative regulators (such as PGRP-LB) expressed by these brain neurons modulate insect oviposition behavior through the regulation of IMD signaling [3]. However, the mechanisms by which IMD signaling in the insect brain regulates oviposition behavior and the pathways through which bacterial PGN in the hemolymph is transferred to brain neurons remain understudied. In *M. musculus*, bacterial PGN derived from the commensal gut microbiota can enter the hemolymph, cross the blood–brain barrier, and reach brain neurons, where some neurons selectively express specific PRRs of the innate immune system to sense PGN. A similar mechanism may exist in insects, but further experiments are needed to confirm this [38].

### 3.3. Effects of Microbes on Insect Resistance to Insecticides

IMD signaling may regulate insect resistance to Bt toxins by affecting MAPK signaling. MAPK signaling acts as a master regulator for the expression of genes related to alkaline phosphatase (ALP) and ATP-binding cassette transporters (ABC transporters) in *Plutella xylostella* [39]. MAPK mediates the transduction of signals from the cell surface to the nucleus, influencing various physiological processes [40]. ALP and ABCC2 serve as receptors for Cry toxins [41]. Studies have shown that the IMD pathway in the midgut of *P. xylostella* is activated during Bt infection [42]. In IMD signaling, TAK1 is a MAP3K, functioning similarly to MAP3K7 [43]. TAK1 can activate downstream MAPKs such as JNK or p38 through different routes [10], which in turn activate relevant transcription factors. These transcription factors not only promote the expression of stress or immune-related genes but may also directly or indirectly regulate the expression of ALP and ABCC genes, thereby affecting insect resistance to Bt toxins. Further experimental validation is needed to confirm these effects (Figure 3).

The production of dual oxidase (DUOX)-dependent reactive oxygen species in the insect gut is regulated by two main signaling pathways. One is DUOX activity signaling, which activates the cadherin 99C/phospholipase C*β*/protein kinase C signaling, leading to intracellular Ca^2+^ release and increased DUOX enzyme activity. The other is DUOX expression signaling, which regulates DUOX expression through mitogen-activated protein kinase kinase kinase 1/IMD–mitogen-activated protein kinase kinase 3–p38 MAPK–activating transcription factor 2 signaling [2]. Inoculation of *Enterococcus* sp. increases the expression of *IMD* and *DUOX* and oxidative stress in *Tribolium castaneum*. It is inferred that the increased oxidative stress mediated by the immune system can reduce tolerance of *T. castaneum* to phosphine [44] (Figure 3).

### 3.4. Effects of Microbes on Insect Chemical Communication

Immune activation can enhance the synthesis of insect pheromones (Figure 3). For example, female *Helicoverpa zea* infected with Hz-2V virus produces increased quantities of sex pheromones, with a pheromone attractiveness that is twice as high as that of uninfected females [45]. Similarly, pheromone production increases in *Apis mellifera* infected with *Nosema* sp. [46]. Further research has shown that *Pseudomonas entomophila* alters lipid metabolism in *D. melanogaster* by activating IMD and IIS, resulting in increased release of fatty-acid derived aggregation pheromones, such as methyl laurate, methyl myristate, and methyl palmitate, which attract healthy *D. melanogaster*. This leads to an increased spread of the pathogen as healthy *D. melanogaster* become infected [47]. This demonstrates the role of IMD–IIS-associated lipid metabolism in the regulation of insect pheromone synthesis.

Immune activation also enhances insect avoidance behavior toward pathogens (Figure 3). When given a choice between food containing two pathogens, mildly pathogenic *Erwinia carotovora* (*Ecc15*) and highly virulent *P. entomophila*, *Drosophila* larvae choose to feed on the food containing *P. entomophila* based on the pathogen odor, rather than choosing based on pathogen virulence. However, after a few minutes or hours, larvae no longer prefer the food containing *P. entomophila* [48]. In insects, the mushroom body (MB) is involved in olfactory learning and regulates innate odor-driven behaviors [49]. *Drosophila* with dysfunctional MB no longer exhibit a preference for food containing specific pathogens.

Further research has revealed that the MB-regulated adaptation behavior of insects toward microbes requires the involvement of octopaminergic neurons [37]. When the expression of PGRP-LC/LE in octopaminergic neurons is downregulated, *D. melanogaster* larvae show a preference for sucrose containing pathogens, and mutants lacking *PGRP-LC/LE* exhibit no preference between two pathogens. These findings suggest that IMD signaling is involved in transmitting pathogen ingestion signals to the nervous system [48]. Pathogen activation of the immune response in *D. melanogaster* induces the expression of PGRP-LC in octopaminergic neurons, which recognize the pathogens [50] and relay the information to the MB, where it lastingly modulates feeding behavior potentially through mechanisms analogous to short-term olfactory associative learning [48]. In insects, PGN can be released into the hemolymph and transferred to other organs [51]. Similarly, in mammals, PGN can be translocated into the brain, where it is sensed by PGRP and subsequently regulates social behavior [38]. Therefore, it is hypothesized that upon bacterial ingestion, PGN travels from the digestive system to the brain of *D. melanogaster* and activates the PGRP-LC signaling in the nervous system [48] (Figure 3).

However, some studies suggest that the avoidance behavior of *D. melanogaster* toward pathogens may be due to the regulation of olfactory discrimination by pathogen infection. When *D. melanogaster* feeds on food contaminated with *Erwinia carotovora carotovora 15* (*Ecc 15*), the gut produces cytokines that activate JAK/STAT signaling in the ensheathing glia of the antennal lobe (AL), inducing the expression of glial monocarboxylate transporters (MCTs) and the lipid binding protein glial lazarillo (Glaz). Glial MCTs promote lipid production in neurons and lipid droplet accumulation in glia by establishing a neuron/glia “lactate shuttle”. Glaz facilitates lipid transport from neurons to glia, affecting neuron/glia metabolic coupling in the AL. This modulates olfactory discrimination, promoting avoidance of bacteria-laced food and increasing host survival [16] (Figure 3).

### 3.5. Effects of Microbes on Insect Cell Turnover

Immune activation promotes both apoptosis and proliferation of insect cells. In aging insect intestines, there is an increase in the total bacterial load, with a dramatic rise in the abundance of Gram-negative *Acetobacteria* within the gut microbiota. To control bacterial load in the gut, IMD signaling becomes excessively activated [52,53]. Overexpression of *IMD*, which activates IMD signaling in *Drosophila*, accelerates apoptosis in fat body cells [54]. IMD signaling activated by bacterial infection induces enterocytes (ECs) shedding into the gut lumen. Further, this shedding is inhibited in *Drosophila* mutants lacking PGRP-LE/IMD/death-related ced-3/nedd2-like protein (Dredd)/Relish function [55]. Additionally, high levels of IMD activation can accelerate the turnover of intestinal epithelial cells and promote compensatory proliferation of intestinal stem cells (ISCs) [56,57]. Activated IMD signaling also enhances gut peristalsis, which helps expel invading bacteria and improves the immune defense of *Drosophila* [58]. However, appropriate negative regulation of immune signaling in non-immune tissues is crucial for normal cell turnover. In *Drosophila* with loss of PGRP-LF function, ectodermal derivative apoptosis is suppressed, leading to the incomplete fusion of the dorsal epidermis and abnormal genitalia rotation [59] (Figure 3).

In addition to IMD signaling, JAK/STAT signaling also promotes insect cell turnover. Infection with *Ecc 15* damages the intestinal epithelial cells, leading to the production of leptin-like (IL-6 family) cytokines known as Unpaireds (Upd, Upd2, and Upd3). These cytokines activate JAK-STAT signaling in ISCs, thereby promoting the proliferation of ISCs and gut epithelial turnover [56,57] (Figure 3).

### 3.6. Effects of Microbes on Insect Lifespan

Activated IMD signaling can extend the lifespan of *Drosophila*. Lifespan is shortened in *Drosophila* with mutations in *PGRP-LC* or *Relish* [52]. Additionally, PGRP-SD enhances the intensity of IMD signaling by antagonizing the extracellular negative regulator PGRP-LB [12]. Overexpression of *PGRP-SD* can prevent dysbiosis of the gut microbiota in *Drosophila*, thereby extending their lifespan. In *PGRP-SD^sk1^* mutant *Drosophila*, increased levels of *Lactiplantibacillus plantarum* in the gut lead to elevated oxidative stress within the insect, resulting in a shortened lifespan [60]. However, excessive activation of IMD signaling can reduce lifespan. In aging *Drosophila* intestines, activation of the transcription factor FoxO inhibits the expression of the negative regulator PGRP-SC2 of IMD signaling, leading to dysregulated Relish/NF-κB activity, which causes stem cell hyperproliferation and epithelial dysplasia. Restoring PGRP-SC2 expression in ECs of the intestinal epithelium can promote tissue homeostasis and extend lifespan [53] (Figure 3).

### 3.7. Effects of Microbes on Insect Sleep

Immune activation can affect sleep in *Drosophila*. For example, systemic infection with *Streptococcus pneumoniae* results in reduced sleep duration and disrupted circadian rhythm in adult *D. melanogaster* [61]. Upregulation of PGRP-LCa in the adult *Drosophila* using the Geneswitch technique, which activates IMD signaling, also leads to a reduction in sleep duration [62]. In contrast, systemic infection with *E. coli* activates an immune response that extends sleep duration in the adult *Drosophila*, and the sleep duration is positively correlated with the level of Relish expression. Sleep duration does not increase in *Relish* mutant *Drosophila* following *E. coli* infection [63]. The current conclusions about whether sleep is induced or suppressed during infection remain controversial. Differences in research outcomes may be attributed to variations in the timing of pathogen infection [64] or differences in the pathogens and their activated immune factors used in the studies [4] (Figure 3).

### 3.8. Metabolic Reprogramming Induced by Microbes

Immune responses are energetically costly. To meet their energy demands, immune cells in insects undergo metabolic reprogramming resembling the Warburg effect observed in mammalian macrophages. The Warburg effect refers to the preference of immune cells for glycolysis to produce lactate and ATP, even in the presence of sufficient oxygen, rather than relying on oxidative phosphorylation (OXPHOS) for ATP production [7].

Immune activation triggers metabolic reprogramming in immune cells (Figure 3). In a resting state, immune cells use the glycolysis-tricarboxylic acid (TCA)–OXPHOS pathway to produce ATP in the most efficient manner (theoretically, 38 ATP molecules per glucose molecule) to meet basal energy demands [65]. Although this method of energy production is highly efficient, the rate of ATP generation is relatively slow and cannot keep up with the rapid energy demands during immune responses. Therefore, immune cells switch to lactate production, which, despite its lower energy yield (only 2 ATP molecules per glucose molecule), provides energy at a much faster rate compared to the glycolysis–TCA–OXPHOS pathway [7].

In mammals, NF-κB signaling mediates metabolic reprogramming induced by hypoxia-inducible factor-1α (HIF-1α). HIF-1α is responsible for initiating cellular metabolic reprogramming in response to hypoxia [66]. The levels of HIF-1α are tightly regulated by oxygen concentration. Without hypoxia, HIF-1α is continuously translated, followed by an immediate hydroxylation by enzyme prolyl hydroxylase dehydrogenase (PHD), which marks HIF-1α for degradation, but activated mammalian Toll-like receptors and NF-κB signaling inhibit PHD through ferritin-mediated signaling, leading to increased expression and stabilization of HIF-1α [67]. High levels of HIF-1α promote glucose uptake, enhance glycolytic and pentose phosphate pathways in immune cells, and suppress the TCA cycle [7].

In *Drosophila*, both genetic and infection-induced activation of Toll and IMD signaling can induce the expression of HIF-1α in immune cells under aerobic conditions. During *S. marcescens* infection in *Drosophila*, IMD/inhibitor of NF-κB kinase (IKK)/Relish signaling induces HIF-1α expression, and knockout of *HIF-1α* increases mortality during infection [68]. Overexpression of the NF-κB factors *Dorsal* or *Cactus* in Toll signaling, or mutations in these genes, also induce HIF-1α expression in *Drosophila* [69]. After immune activation in *Heliothis virescens* larvae, transcriptome analysis of hemocytes reveals increased expression of glycolytic genes and lactate dehydrogenase gene [70]. During the parasitoid infestation of *Drosophila* larvae, increased proliferation and differentiation of specialized immune cells called lamellocytes is associated with a hemocyte-specific increase in the expression of glycolytic genes, accompanied by increased glucose consumption and lactate production [71]. Although insect immune cells significantly increase glucose consumption, glycolysis, and lactate production upon activation, there are still substantial metabolic differences between insects and mammals, necessitating further detailed characterization of metabolic changes in insects.

Immune activation also impacts systemic metabolism in insects (Figure 3). During immune response, the uptake of glucose by immune cells can influence systemic metabolism. For example, during parasitoid wasp infection, *Drosophila* larvae activate immune cells that require substantial glucose consumption [71]. In this context, immune activation significantly inhibits other host metabolic processes during infection, and thus infection with parasitoid wasps and *Listeria monocytogenes* reduces triglyceride and glycogen reserves in *D. melanogaster* [32,72].

Further research has identified adenosine (Ado) as a mediator of the systemic metabolic switch during immune activation in insects. Ado, at the cost of depleting energy reserves and inhibiting systemic metabolism, provides immune cells with the required energy either for their rapid proliferation and the differentiation of lamellocytes and effective encapsulation of parasitoid eggs, or for their effective phagocytosis of bacteria [7]. Hemocytes in *Drosophila* hemolymph recognize parasitoid eggs laid by wasps and activate the proliferation and differentiation of specialized immune cells called lamellocytes, which encapsulate and destroy the parasitoid eggs. Activated lamellocytes increase glycolysis and glucose consumption, and they usurp glucose from other tissues through Ado release. Extracellular Ado suppresses metabolism in other tissues via the adenosine receptor. By regulating systemic metabolism through Ado release, the immune system gains priority in energy usage [71].

## 4. Outlook

Increasing evidence confirms that immune signaling serves as a coordinator between insect defense, physiology, and behavior. However, the functional characteristics of downstream genes regulated by immune signaling remain unclear. Consequently, understanding how immune signaling regulates non-immune physiological activities such as growth and development, reproduction, pesticide resistance, chemical communication, cell turnover, lifespan, and sleep is still in its early stages. To elucidate the mechanisms by which immune signaling regulates insect physiology and behavior, the following key questions need to be addressed: (1) How does IMD signaling interact with IIS to regulate insect oogenesis? (2) Does IMD signaling regulate the expression of Bt receptors such as ALP and ABC transporters via MAPK signaling, thereby altering insect resistance to Bt? (3) How does IMD signaling interact with IIS to regulate lipid metabolism and subsequently affect the synthesis of insect pheromones? (4) Does IMD and Toll signaling mediate metabolic reprogramming of insect immune cells and a systemic metabolic switch that are induced by HIF-1α?

To investigate the impact of microbes on insect hosts mediated by the immune system, oral infection, systemic infection, and genetic manipulation are usually adopted to activate the insect immune system. Oral infection activates the insect immune system thorough ingestion of pathogens via the digestive tract, which simulates natural infection processes and is commonly used to study gut immunity in insects. For example, oral infection with *Ecc15* leads to changes in *Relish*-dependent gene expression in the midgut, including immune and metabolic gene regulators [56]. Systemic infection triggers a systemic immune response through introduction of pathogens into the insect body via microinjection. For instance, systemic infection with *Micrococcus luteus* results in reduced Akt phosphorylation and triglyceride content in the *Drosophila* fat body [22]. Genetic manipulation uses biotechnological techniques (such as gene knockout, overexpression, or gene editing like UAS-Gal4, CRISPR/Cas9) to modify insect genes, activating or inhibiting specific immune responses. This method is often used to study the functions of specific genes in the immune system or their effects on other physiological functions. For example, overexpression of *Toll* in the *Drosophila* fat body using UAS-Gal4 suppresses Akt phosphorylation and reduces triglyceride content [19].

Although research on the interaction between immunity and metabolism in insects has lagged compared to mammalian systems, recent studies have revealed that the mechanisms of immune and metabolic interactions during immune responses are highly similar between insects and mammals. Therefore, knowledge about the metabolic reprogramming of immune cells and a systemic metabolic switch induced by immune cell activation in mammals provides valuable insights for in-depth studies in insects. Additionally, advancements in metabolomics analysis techniques facilitate related research [73]. For instance, matrix-assisted laser desorption ionization coupled with mass spectrometry imaging allows visualization of the spatial distribution of metabolites within intact tissue samples, showing great potential in cell-specific metabolomics research [74]. Several insect infection models, such as *D. melanogaster*, *Bombyx mori*, and *Anopheles stephensi* [5], have been established. Especially, *D. melanogaster* has a simple diet and a short life cycle, and mutants are available for many genes, which makes it an excellent model for studying inter-tissue communication [75]. They can be used to investigate the regulation of systemic metabolism during immune responses and inter-tissue communication, revealing interactions between immunity and metabolism.

## Data Availability

Not applicable.
